# From cow to cheese: genetic parameters of the flavour fingerprint of cheese investigated by direct-injection mass spectrometry (PTR-ToF-MS)

**DOI:** 10.1186/s12711-016-0263-4

**Published:** 2016-11-16

**Authors:** Matteo Bergamaschi, Alessio Cecchinato, Franco Biasioli, Flavia Gasperi, Bruno Martin, Giovanni Bittante

**Affiliations:** 1 Department of Agronomy, Food, Natural Resources, Animals and Environment (DAFNAE), University of Padua, Viale dell’Università 16, 35020 Legnaro, PD Italy; 2 Department of Food Quality and Nutrition, Research and Innovation Centre, Fondazione Edmund Mach (FEM), Via E. Mach 1, 38010 San Michele all’Adige, TN Italy; 3 INRA, UMR Herbivores, 63122 Saint-Genès Champanelle, France; 4 Clermont Université, VetAgro Sup, BP 10448, 63000 Clermont-Ferrand, France

## Abstract

**Background:**

Volatile organic compounds determine important quality traits in cheese. The aim of this work was to infer genetic parameters of the profile of volatile compounds in cheese as revealed by direct-injection mass spectrometry of the headspace gas from model cheeses that were produced from milk samples from individual cows.

**Methods:**

A total of 1075 model cheeses were produced using raw whole-milk samples that were collected from individual Brown Swiss cows. Single spectrometry peaks and a combination of these peaks obtained by principal component analysis (PCA) were analysed. Using a Bayesian approach, we estimated genetic parameters for 240 individual spectrometry peaks and for the first ten principal components (PC) extracted from them.

**Results:**

Our results show that there is some genetic variability in the volatile compound fingerprint of these model cheeses. Most peaks were characterized by a substantial heritability and for about one quarter of the peaks, heritability (up to 21.6%) was higher than that of the best PC. Intra-herd heritability of the PC ranged from 3.6 to 10.2% and was similar to heritabilities estimated for milk fat, specific fatty acids, somatic cell count and some coagulation parameters in the same population. We also calculated phenotypic correlations between PC (around zero as expected), the corresponding genetic correlations (from −0.79 to 0.86) and correlations between herds and sampling-processing dates (from −0.88 to 0.66), which confirmed that there is a relationship between cheese flavour and the dairy system in which cows are reared.

**Conclusions:**

This work reveals the existence of a link between the cow’s genetic background and the profile of volatile compounds in cheese. Analysis of the relationships between the volatile organic compound (VOC) content and the sensory characteristics of cheese as perceived by the consumer, and of the genetic basis of these relationships could generate new knowledge that would open up the possibility of controlling and improving the sensory properties of cheese through genetic selection of cows. More detailed investigations are necessary to connect VOC with the sensory properties of cheese and gain a better understanding of the significance of these new phenotypes.

**Electronic supplementary material:**

The online version of this article (doi:10.1186/s12711-016-0263-4) contains supplementary material, which is available to authorized users.

## Background

Volatile organic compounds (VOC) are important molecules that determine the distinct flavours of cheeses and, consequently, their perceived quality [1, 2]. The development of flavour in cheese depends on the origin and gross composition of milk [3]. Milk provides the main components for the cheese-making process as well as micro-organisms that release proteases and lipases, which catalyse the breakdown of lipids and proteins and lead to flavour development in cheese [4]. It is well known that cheese types are characterized by different aroma profiles [5, 6] and several studies have focused on the relationships between the sensory properties of cheese and the dairy system used, the cows’ feeding regime and milk quality [7–9]. Moreover, sensory appraisal can have a huge impact on the economic value of cheese [10, 11]. Given the subjectivity, high cost and limited repeatability of sensory evaluation, and the need to better understand its chemical and biological basis, in recent years several techniques have been used to determine the qualitative characteristics of cheese flavour compounds [12–14]. Gas-chromatography combined with headspace extraction has been commonly used to investigate the link between VOC and the flavour of cheese [15–17]. Solid-phase micro-extraction and gas-chromatography mass spectrometry have been used to extract VOC from individual full-fat ripened cheeses in order to study the effects of dairy system, herd, and the cows’ parity, stage of lactation and milk yield on these quality traits [18]. Recently, a model cheese procedure was used to produce a large number (more than 1000) of individual model cheeses [19] that were used to estimate the genetic parameters of cheese yields and nutrient recovery [20]. In addition, the direct-injection spectrometry method (proton transfer reaction-time of flight-mass spectrometry, PTR-ToF-MS) was used for the first time to obtain the fingerprints of volatile compounds in the same model cheeses [21]. Two hundred and forty peaks were detected from which the principal components (PC) were extracted which showed that dairy systems and individual cow characteristics had an effect on these new phenotypes.

In spite of the centrality and importance of VOC, which are potentially related to sensory properties, to date, no research has been carried out to estimate the heritability and genetic correlations of their concentrations in cheese. Given the economic importance of the perceived flavour in the cheese industry, a detailed knowledge of the genetic parameters of the VOC profile is fundamental to be able to evaluate the possibility of modifying cheese flavour in the future through breeding programmes using direct or indirect prediction of these traits (e.g., using infrared technology). Our objective was to estimate the genetic parameters of spectrometry peaks obtained by PTR-ToF-MS and of their PC to characterize the volatile compound fingerprint of model cheeses obtained from the milk of individual Brown Swiss cows.

## Methods

### Field data

This work is a part of the “Cowability-Cowplus projects”, which involve collection of milk samples from a large number of Brown Swiss cows (n = 1075) from different herds (n = 72) located in northern Italy (Trento province). The production environment was previously described in [22]. On each day, only one herd was visited and 15 cows from the herd were individually sampled once during evening milking. The herds were sampled over a full year to cover all seasons and rearing conditions. In the experimental area, cows on the permanent farms are not grazed and their feeding regime is almost constant all year around. Part of the herds are moved to Alpine pastures during summer, but samples were not taken from them during transhumance. Detailed descriptions of the herds, the cows’ characteristics, and the sampling procedure are available in previous papers on cheese VOC [18, 21]. Briefly, milk samples (without preservative) were immediately refrigerated (4 °C) and transferred to the Cheese Making Laboratory of the Department of Agronomy, Food, Natural Resources, Animals and Environment (DAFNAE) of the University of Padua (Legnaro, Padua, Italy). All milk samples were collected as routine collection and thus no ethical approval was necessary. Data on individual cows and herds were provided by the Superbrown Consortium of Bolzano and Trento (Italy), and pedigree information was supplied by the Italian Brown Swiss Cattle Breeders Association (ANARB, Verona, Italy). The analysis included cows with phenotypic records on the investigated traits and all their known ancestors. Each sampled cow had at least four generations of known ancestors, and the pedigree file included 8845 animals. There were 1326 sires in the whole pedigree, among which 264 had progeny with records in the dataset (each sire had between 2 and 80 daughters).

### Individual cheese-making procedure

Gross milk composition was measured using a MilkoScan FT6000 (Foss Electric A/S, Hillerød, Denmark). Somatic cell count was obtained from the Fossomatic FC counter (Foss) then converted to somatic cell score (SCS) by logarithm transformation [23]. All raw whole-milk samples were transformed into cheeses within 20 h of collection. The cheese-making procedure was designed to produce a laboratory “model-cheese” under the normal laboratory conditions for testing the coagulation properties of milk [19]. Briefly, 1500 mL of milk were heated at 35 °C in a stainless steel micro-vat, to which was added a thermophilic starter culture to reduce the effects of the microflora of the milk samples, and then rennet. On average, milk rennet coagulation time (RCT) was 20.3 min. Commercial rennet [Hansen standard 160 with 80 ± 5% chymosin and 20 ± 5% pepsin; 160 international milk clotting units (IMCU) × mL^−1^; Pacovis Amrein AG, Bern, Switzerland] was diluted 20:1 with distilled water, and 9.6 mL of rennet solution was added to each vat to obtain a final concentration of 51.2 IMCU × L^−1^ of milk. The resulting curd from each vat was cut, drained, shaped into wheels, pressed, salted and weighed. All model cheeses were ripened for 60 days at 15 °C before sampling for the VOC analyses.

Descriptive statistics on daily milk yield and fat and protein content of milk from the Brown Swiss cows selected for the study, and fat and protein content of the model cheeses are in Table 1.

**Table 1:** Descriptive statistics for milk production, cheese composition and the first principal components characterizing the volatile compound fingerprint of 1075 individual model cheeses analysed by PTR-ToF-MS

Traits	Mean	CV (%)
Milk yield (kg × day^−1^)	24.6	32.1
Milk composition		
Fat (%)	4.4	20.5
Protein (%)	3.8	10.5
Fat/protein	1.18	21.2
Casein/protein	0.769	2.34
SCS (U)	3.03	1.86
Cheese composition		
Fat (%)	38.2	11.5
Protein (%)	27.1	15.1
Cheese volatile fingerprint	Total phenotypic variance (%)	Cumulative phenotypic variance (%)
PC1	28.30	28.30
PC2	10.90	39.20
PC3	8.59	47.79
PC4	7.61	55.40
PC5	6.06	61.46
PC6	3.74	65.19
PC7	2.68	67.87
PC8	2.26	70.14
PC9	1.85	71.98
PC10	1.58	73.56

SCS = log_2_(SCC/100,000) + 3, where SCC is somatic cells per mL

### PTR-ToF-MS analysis

A cylindrical sample (1.1 × 3.5 cm) of each cheese was kept at −80 °C until VOC analysis. The headspace gas of each model cheese (n = 1075) was measured using a commercial PTR-ToF-MS 8000 instrument supplied by Ionicon Analytik GmbH, Innsbruck (Austria) following a modified version of the procedure described in [24]. Details of the analytical procedures and peak selection are in [18]. Briefly, cheese samples chosen randomly from the set of 1075 samples were thawed and kept at room temperature (about 20 °C) for 6 h. Sub-samples (3 g) from each cheese were placed in glass vials (20 mL; Supelco, Bellefonte, USA) equipped with PTFE/Silicone septa (Supelco) and were measured every day. Internal calibration and peak extraction were performed as described in [25], which made it possible to assign, in some cases, a chemical formula to relevant spectrometry peaks. Absolute headspace VOC concentrations, expressed as parts per billion by volume (ppb_v_), were calculated from peak areas using the formula described in the literature [26] with a constant reaction rate coefficient of the proton transfer reaction of 2 × 10^−9^ cm^3^/s.

### PTR-ToF-MS data

As discussed in detail in [21], 619 peaks describing VOC were obtained from the headspace gas of 1075 individual model cheeses using PTR-ToF-MS. Data compression was performed by selecting the peaks that displayed a spectrometry area greater than 1 part per billion by volume, which yielded 240 peaks after elimination of interfering ions. In addition, tentative interpretation of the spectrometry peaks was made based on the fragmentation patterns of the 61 most important volatile compounds in terms of spectrometry area that were retrieved from the available solid-phase micro-extraction gas chromatography mass spectrometry data on the same model cheeses, or from the literature, representing about 80% of the total spectral intensity. The strongest peaks detected by PTR-ToF-MS were at *m*/*z* 43.018 and 43.054, tentatively attributed to alkyl fragments, and at *m*/*z* 61.028 and 45.033, tentatively attributed to acetic acid and ethanol, respectively [18, 21].

### Multivariate analysis of VOC

Multivariate data treatment (PCA) was carried out on the standardized spectrometry peaks using Statistica 7.1 (StatSoft, Paris) in order to summarize the information and provide a new set of ten PC. The statistical methodology is described in detail in [21]. The descriptive statistics of these ten PC, which represented 73.6% of the total variance of all VOC, are in Table 1.

### Genetic parameters of VOC and their PC

Non-genetic effects analysed in a previous phenotypic study on the same dataset [21] were considered for the estimation of the genetic parameters of VOC and of their PC, but the effects of the micro-vats that were used on each sampling-processing date were not included in the statistical model because the adopted model cheese-making procedure showed good repeatability and reproducibility [19, 21].

All genetic models accounted for the effects of herd/sampling-processing date (72 levels) and the cows’ days in milk (DIM; class 1: <50 days, class 2: 51–100 days, class 3: 101–150 days; class 4: 151–200 days; class 5: 201–250 days; class 6: 251–300 days; class 7: >300 days) and parity (1–4 or more) for all traits.

Univariate models were fitted to estimate variance components and heritabilities for the traits analyzed.

The model assumed for VOC and PC was:

1 $${\mathbf{y}} = {\mathbf{Xb}} + {\mathbf{Z}}_{ 1} {\mathbf{h}} + {\mathbf{Z}}_{ 2} {\mathbf{a}} + {\mathbf{e}},$$

where **y** is the vector of phenotypic records with dimension *n*; **X**,** Z**_1_, and** Z**_2_ are appropriate incidence matrices for systematic effects **b,** herd/sampling-processing date effects **h**, and polygenic additive genetic effects **a**, respectively; and **e** is the vector of residual effects. More specifically, **b** included the non-genetic effects of DIM and parity.

All models were analysed using a standard Bayesian approach. Joint distribution of the parameters in a given model was proportional to:

$$\begin{aligned} & p\left( {{\mathbf{b}},{\mathbf{h}},{\mathbf{a}},\sigma_\text{e}^{2} ,\sigma_\text{h}^{2} ,\sigma_\text{a}^{2} |{\mathbf{y}}} \right) \propto p\left( {{\mathbf{y}}|{\mathbf{b}},{\mathbf{h}},{\mathbf{a}},\sigma_\text{e}^{2} } \right)p\left( {\sigma_\text{e}^{2} } \right)p\left( {\mathbf{b}} \right) \\ & \quad \times p\left( {{\mathbf{h}}|\sigma_\text{h}^{2} } \right)p\left( {\sigma_\text{h}^{2} } \right)p\left( {{\mathbf{a}}|{\mathbf{A}},\sigma_\text{a}^{2} } \right)p\left( {\sigma_\text{a}^{2} } \right), \\ \end{aligned}$$

where **A** is the numerator relationship matrix between individuals, and $\sigma_\text{e}^{2},$ $\sigma_\text{h}^{2}$ and $\sigma_\text{a}^{2}$ are the residual, herd/sampling-processing date and additive genetic variances, respectively. The a priori distribution of **h** and **a** were assumed to be multivariate normal, as follows:

$$p\left( {{\mathbf{h}}|\sigma_\text{h}^{2} } \right)\sim\,N\left( {{\mathbf{0}},{\mathbf{I}}\sigma_\text{h}^{2} } \right),$$

$$p\left( {{\mathbf{a}}|\sigma_\text{a}^{2} } \right)\sim\,N\left( {{\mathbf{0}},{\mathbf{A}}\sigma_\text{a}^{2} } \right),$$

where **I** is an identity matrix with dimensions equal to the number of elements in **h**. Flat priors were assumed for **b** and the variance components.

To estimate the genetic correlations between VOC, PC and milk composition, we conducted a set of bivariate analyses that implemented model (1) in its multivariate version. In this case, the traits involved were assumed to jointly follow a multivariate normal distribution along with the additive genetic, herd and residual effects. The corresponding prior distributions of these effects were:

$${\mathbf{a}}|{\mathbf{G}}_{0} ,{\mathbf{A}}\sim\, MVN\left( {0,{\mathbf{G}}_{0} , \otimes {\mathbf{A}}} \right),$$

$${\mathbf{h}}|{\mathbf{H}}_{0} , \sim\,N\left( {0,{\mathbf{H}}_{0} , \otimes {\mathbf{I}}_{n} } \right),$$

$${\rm and}\quad {\mathbf{e}}|{\mathbf{R}}_{0} ,\sim\,N\left( {0,{\mathbf{R}}_{0} , \otimes {\mathbf{I}}_{m} } \right),$$

where **G**_0_, **H**_0_ and **R**_0_ are the corresponding variance–covariance matrices between the involved traits, and **a**, **h** and **e** are vectors with dimensions equal to the number of animals in the pedigree (*n* and *m*) times the number of traits considered.

### Bayesian inference

Marginal posterior distributions of all unknowns were estimated using the Gibbs sampling algorithm [27]. The TM program (http://snp.toulouse.inra.fr/~alegarra) was used for all Gibbs sampling procedures. Chain lengths and burn-in period were assessed by visual inspection of the trace plots and by the diagnostic tests described in [28, 29]. After preliminary analysis, chains of 850,000 samples were used, with a burn-in period of 50,000. One in every 200 successive samples was retained. The lower and upper bounds of the highest 95% probability density regions (HPD 95%) for the parameters of concern were obtained from the estimated marginal densities. The posterior mean was used as the point estimate for all parameters.

Across-herd heritability was computed as:

$${\text{h}}_{\text{AH}}^{2} = \frac{{\upsigma_{\text{a}}^{2}}}{{\upsigma_{\text{a}}^{2} + \upsigma_{\text{h}}^{2} + \upsigma_{\text{e}}^{2}}},$$

where $\upsigma_{\text{a}}^{2}$, $\upsigma_{\text{h}}^{2}$, and $\upsigma_{\text{e}}^{2}$ are additive genetic, herd/sampling-processing date and residual variances, respectively.

Intra-herd heritability was computed as:

$${\text{h}}_{\text{IH}}^{2} = \frac{{\upsigma_{\text{a}}^{2}}}{{\upsigma_{\text{a}}^{2} + \upsigma_{\text{e}}^{2}}},$$

where $\upsigma_{\text{a}}^{2}$ and $\upsigma_{\text{e}}^{2}$ are additive genetic and residual variances, respectively.

Additive genetic correlations (*r*_*a*_) were computed as:

$$r_{a} = \frac{{\upsigma_{{{\text{a}}1,{\text{a}}2}}}}{{\upsigma_{{{\text{a}}1}} \cdot \upsigma_{{{\text{a}}2}}}},$$

where $\upsigma_{{{\text{a}}1,{\text{a}}2}}$ is the additive genetic covariance between traits 1 and 2, and $\upsigma_{{{\text{a}}1}}$ and $\upsigma_{{{\text{a}}2}}$ are the additive genetic standard deviations for traits 1 and 2, respectively.

The herd/sampling-processing date correlations (*r*_*h*_) were computed as:

$$r_{h} = \frac{{\upsigma_{{{\text{h}}1,{\text{h}}2}}}}{{\upsigma_{{{\text{h}}1}} \cdot \upsigma_{{{\text{h}}2}}}},$$

where $\upsigma_{{{\text{h}}1,{\text{h}}2}}$ is the herd/sampling-processing date covariance between traits 1 and 2, and $\upsigma_{{{\text{h}}1}}$ and $\upsigma_{{{\text{h}}2}}$ are the herd/sampling-processing date standard deviations for traits 1 and 2, respectively.

The residual correlations (*r*_*e*_) were computed as:

$$r_{e} = \frac{{\upsigma_{{{\text{e}}1,{\text{e}}2}}}}{{\upsigma_{{{\text{e}}1}} \cdot \upsigma_{{{\text{e}}2}}}}$$

where $\upsigma_{{{\text{e}}1,{\text{e}}2}}$ is the residual covariance between traits 1 and 2, and $\upsigma_{{{\text{e}}1}}$ and $\upsigma_{{{\text{e}}2}}$ are the residual standard deviations for traits 1 and 2, respectively.

## Results and discussion

### Variance components and heritability of individual spectrometry peaks of the volatile compound fingerprint of cheese

A univariate Bayesian animal model was applied to each of the 240 individual spectrometry peaks. The variance components and heritability estimates are in Table S1 (see Additional file 1: Table S1). Table 2 shows the distribution of the intra-herd heritability estimates for the individual peaks related to the VOC of the cheese samples. Only a few peaks are characterized by a very low heritability (six peaks with a heritability lower than 3.5%).

**Table 2:** Average concentrations and estimates of phenotypic ($\upsigma_{\text{P}}$), residual ($\upsigma_{\text{E}}$), herd ($\upsigma_{\text{H}}$), and additive genetic ($\upsigma_{\text{A}}$) standard deviations, and of intra-herd heritability (h^2^) categories for 240 spectrometric peaks from PTR-ToF-MS analysis of 1075 individual model cheeses made from Brown Swiss cows’ milk

h^2^ (%)	Peaks numbers	Concentration^a^		Average of the SD				Average h^2^
		Average ln ppb_v_^b^	CV (%)^c^	$\upsigma_{\text{P}}$	$\upsigma_{\text{E}}$	$\upsigma_{\text{H}}$	$\upsigma_{\text{A}}$	
<2	0							
2–4	11	6.26	29.1	1.008	0.874	0.468	0.166	0.035
4–6	60	6.25	28.3	1.006	0.823	0.480	0.188	0.050
6–8	69	6.05	31.4	1.004	0.864	0.402	0.234	0.068
8–10	38	5.30	20.5	1.004	0.817	0.459	0.253	0.087
10–12	22	5.19	22.1	1.006	0.795	0.450	0.276	0.108
12–14	20	6.73	27.8	0.995	0.725	0.579	0.280	0.130
14–16	13	5.28	12.3	0.994	0.795	0.488	0.328	0.146
16–18	4	4.91	5.2	1.001	0.788	0.498	0.358	0.172
18–20	1	5.17		0.986	0.721	0.572	0.352	0.192
>20	2	5.33	1.2	1.020	0.666	0.691	0.344	0.211
All	240	5.90	28.5	1.003	0.822	0.462	0.239	0.082

*SD* standard deviation

^a^Mean value of each peak of the various classes

^b^Data expressed in natural log-transformed (ln) parts per billion by volume

^c^Coefficient of variation of the mean value of each peak calculated by dividing the standard deviation by the mean of the ppb_v_ concentration of the PTR spectrometry peaks within each intra-herd heritability class

Table 2 shows that there is a tendency towards a decrease in concentration with increasing heritability (note that the concentration is expressed on a logarithmic scale). This can be interpreted as a decrease in primary substrates, which are involved in a large number of potential metabolic pathways involved in the production of VOC. Compounds with lower concentrations are sometimes characterized by a proportional increase in instrumental error and, then, a decrease in their heritability is expected. This is not true for the spectrometry peaks that were examined in this study, although a large number of peaks with very low concentrations (<1 ppb_v_) were not included here. This is an indirect indication of the enormous potential of the PTR-ToF-MS method for evaluating the volatile compound fingerprint of cheese. The ten VOC that had the highest estimated heritability among the VOC that were tentatively identified or unidentified are in Tables 3 and 4, respectively. The results confirm that several individual spectrometry peaks are characterized by heritability estimates of the same magnitude as those for milk yield, some milk quality traits [30, 31] and also some technological parameters [32].

**Table 3:** Spectrometry peaks with the highest heritability (h^2^) with tentative identification of volatile compounds from PTR-ToF-MS analysis of 1075 model cheeses; their phenotypic ($\upsigma_{\text{P}}$), residual ($\upsigma_{\text{E}}$), herd ($\upsigma_{\text{H}}$) and additive genetic ($\upsigma_{\text{A}}$) SD

Measured mass (*m*/*z*)	Theoretical mass (*m*/*z*)	Tentative identification	Sum formula	ln ppb_v_^a^	CV (%)	SD				h^2^
						$\upsigma_{\text{P}}$	$\upsigma_{\text{E}}$	$\upsigma_{\text{H}}$	$\upsigma_{\text{A}}$	
49.011	49.0106	Methanethiol	CH_5_O^+^	6.82	9.8	0.994	0.800	0.507	0.302	0.125
57.033	57.0335	3-Methyl-1-butanol	C_3_H_5_O^+^	6.70	10.0	1.007	0.805	0.516	0.317	0.134
75.080	75.0810	Butan-1-ol, pentan-1-ol, heptan-1-ol	C_4_H_11_O^+^	7.70	14.7	0.990	0.763	0.554	0.302	0.136
81.070	81.0699	Alkyl fragment (terpenes)	C_6_H_9_^+^	5.37	8.6	1.021	0.669	0.692	0.340	0.206
83.086	83.0855	Hexanal, nonanal	C_6_H_11_^+^	6.13	11.3	0.998	0.825	0.453	0.333	0.140
95.017	95.0161	Methyldisulfanylmethane	C_2_H_7_O_2_S^+^	5.04	14.1	1.013	0.847	0.415	0.370	0.161
117.091	117.0910	Ethyl butanoate, ethyl-2-methylpropanoate	C_6_H_13_O_2_^+^	9.14	6.6	0.986	0.780	0.526	0.295	0.125
118.095	118.0940	Ethyl butanoate, ethyl-2-methylpropanoate	C5^[13]^CH_13_O_2_^+^	6.53	8.7	0.986	0.775	0.531	0.298	0.129
145.123	145.1220	Ethyl hexanoate, octanoic acid	C_8_H_17_O_2_^+^	7.43	10.5	0.971	0.777	0.495	0.304	0.133
146.126	146.1260	Ethyl hexanoate, octanoic acid	C_7_^[13]^CH_17_O_2_^+^	5.30	11.5	0.972	0.774	0.498	0.310	0.138

*SD* standard deviation

^a^Data expressed in natural log-transformed (ln) parts per billion by volume

**Table 4:** Unidentified spectrometry peaks with the highest heritability (h^2^) from PTR-ToF-MS analysis of 1075 model cheeses; their phenotypic ($\upsigma_{\text{P}}$), residual ($\upsigma_{\text{E}}$), herd ($\upsigma_{\text{H}}$) and additive genetic ($\upsigma_{\text{A}}$) SD

*m*/*z*	ln ppb_v_^a^	CV (%)	SD				h^2^
			$\upsigma_{\text{P}}$	$\upsigma_{\text{E}}$	$\upsigma_{\text{H}}$	$\upsigma_{\text{A}}$	
50.056	4.58	9.6	0.992	0.754	0.562	0.313	0.147
93.431	4.54	10.0	0.998	0.759	0.542	0.354	0.179
95.095	5.44	18.1	0.991	0.766	0.534	0.331	0.157
111.104	5.08	18.5	0.991	0.774	0.528	0.322	0.147
120.092	4.95	14.5	0.998	0.797	0.478	0.363	0.172
121.122	5.17	15.8	0.986	0.721	0.572	0.352	0.192
136.140	4.26	8.9	0.990	0.759	0.549	0.319	0.150
137.132	5.28	7.3	1.019	0.663	0.691	0.348	0.216
171.173	4.87	15.7	1.004	0.905	0.202	0.384	0.152
173.153	5.20	9.3	0.997	0.825	0.440	0.345	0.149

*SD* standard deviation

^a^Data expressed in natural log-transformed (ln) parts per billion by volume

It is interesting to note that some peaks are related to PC and correspond with specific odours and aromas detected in many cheese varieties [2, 13]. For instance, among the masses that were most positively correlated with PC, Bergamaschi et al. [21] detected *m*/*z* 117.091 and *m*/*z* 145.123, and their isotopes *m*/*z* 118.095 and 146.126, which in our study had heritabilities of 12 and 13%, respectively. The same authors tentatively attributed these peaks to ethyl butanoate, ethyl-2-methylpropanoate and ethyl hexanoate [21]. Esters originate from the interaction between free fatty acids and alcohols that are produced by microorganisms and are responsible for fruity-floral notes in cheese aroma [13]. In addition, the peak with a theoretical mass *m*/*z* 95.017 that is associated with methyldisulfanylmethane had a heritability of 12.5% and characterized PC4 (Table 3). This sulphur compound is either derived from the diet or formed from the amino acid methionine that is released during cheese ripening [3]. We found that *m*/*z* 81.070, which is associated with terpene fragments, had a relatively high heritability (20.6%). Terpenes are products of the degradation of carotenoids [33] and are listed as cheese odorants that have a fresh, green odour [34]. It is well known that volatile terpene in cheese is a biomarker of the area of production, and the type and phenological stage of forage [35, 36]. We found that the amount of these molecules is also affected by the genetic background of the animals (Table 3). As discussed by Bugaud et al. [37], the abundance of plants such as *Gramineae* or dicotyledons can influence the concentration of plasmin and terpenes in milk.

These particular peaks should be the first to be studied in terms of their effect on the flavour and acceptability of cheese, because they display exploitable genetic variation.

### Variance components and heritability of PC extracted from volatile profiles of cheese

The proportions of variance explained by the first ten PC are in Table 1. A list of the tentatively identified individual VOC that were found as the most highly correlated with each PC is in Additional file 2: Table S2. Estimates of the marginal posterior densities for the additive genetic, herd/sampling-processing date and residual variances are in Table 5. The herd/sampling-processing date variance was always larger than the genetic variance of each PC, but was smaller than the residual variance in all but two cases i.e. PC3 had a greater herd/sampling-processing date variance than the residual variance, while for PC5 they were of the same magnitude. These data show that the proportions of the main sources of variation in individual PC (genetic, herd and individual/residual components) differ.

**Table 5:** Features of marginal posterior densities of additive genetic ($\upsigma_{\text{A}}^{2}$), herd/sampling-processing date ($\upsigma_{\text{H}}^{2}$), and residual ($\upsigma_{\text{E}}^{2}$) variances, and across-herd (${\text{h}}_{\text{AH}}^{2}$) and intra-herd (${\text{h}}_{\text{IH}}^{2}$) heritabilities for principal components derived from the volatile fingerprint of 1075 individual model cheeses analysed by PTR-ToF-MS

Traits	$\upsigma_{\text{A}}^{2}$		$\upsigma_{\text{H}}^{2}$		$\upsigma_{\text{E}}^{2}$		${\text{h}}_{\text{AH}}^{2}$		${\text{h}}_{\text{IH}}^{2}$	
	Mean	HPD 95%	Mean	HPD 95%	Mean	HPD 95%	Mean	HPD 95%	Mean	HPD 95%
PC1	4.49	0.46; 11.70	15.14	9.79; 22.59	48.81	41.85; 55.09	0.065	0.01; 0.17	0.084	0.01; 0.21
PC2	1.48	0.09; 4.04	9.12	6.21; 13.24	15.95	13.53; 18.04	0.056	0.01; 0.15	0.085	0.01; 0.22
PC3	0.71	0.05; 1.90	11.77	8.22; 16.60	9.31	8.09; 10.44	0.033	0.01; 0.08	0.071	0.01; 0.18
PC4	1.62	0.15; 3.96	2.80	1.69; 4.37	14.27	12.05; 16.28	0.086	0.01; 0.21	0.102	0.01; 0.24
PC5	0.43	0.02; 1.27	7.30	5.08; 10.43	7.21	6.32; 8.04	0.029	0.01; 0.09	0.057	0.01; 0.16
PC6	0.22	0.01; 0.73	2.93	1.97; 4.23	5.97	5.36; 6.59	0.024	0.01; 0.08	0.036	0.01; 0.11
PC7	0.47	0.04; 1.27	1.61	1.06; 2.39	4.50	3.77; 5.11	0.071	0.01; 0.19	0.095	0.01; 0.25
PC8	0.48	0.06; 1.14	0.64	0.36; 1.04	4.43	3.79; 5.04	0.086	0.01; 0.20	0.098	0.01; 0.23
PC 9	0.21	0.01; 0.65	0.47	0.25; 0.77	3.86	3.40; 4.30	0.047	0.01; 0.16	0.053	0.01; 0.14
PC10	0.30	0.01; 0.86	0.47	0.26; 0.76	3.08	2.56; 3.50	0.078	0.01; 0.22	0.089	0.01; 0.25

Mean = mean of the marginal posterior density of the parameter; HPD 95% = lower and upper bound of the 95% highest posterior density region

**Fig. 1:**
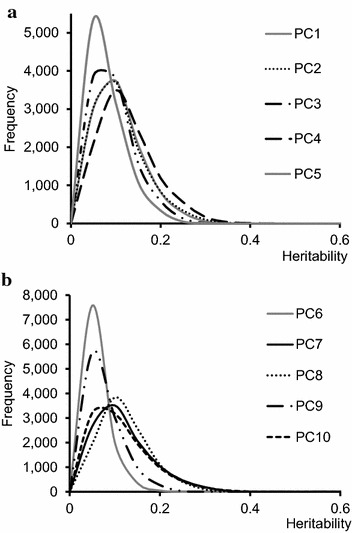
Marginal posterior distributions of the intra-herd heritability for principal components PC1 to PC5 (**a**) and PC6 to PC10 (**b**). Principal components derived from the volatile fingerprint of 1075 individual model cheeses analysed by PTR-ToF-MS

All ten PC that were extracted from the volatile compound fingerprint of the model cheeses had a heritability higher than 0 (Table 5). Across-herd heritability of the PC ranged from 2.4 to 8.6%, while intra-herd heritability ranged from 3.6 to 10.2%. The marginal posterior distributions of the intra-herd heritability of these ten PC are in Fig. 1a, b.

Notably, the two most important PC, which explained about 40% of the overall variability, were characterized by similar heritabilities (${\text{h}}_{\text{IH}}^{2}$: 8.4% for PC1 and 8.5% for PC2).

Herds were previously classified according to dairy system, i.e. traditional or modern, which vary in terms of milk yield, destination of milk, facilities, feed management and use of maize silages [21, 22]. Modern dairy farms had modern facilities, loose animals, milking parlours and used total mixed rations with or without silage, whereas traditional dairy farms had small buildings, animals were tied and milked at the stall, and the feed was mainly composed of hay and compound feed. It is worth noting that PC1 was not affected by dairy system, while PC2 was higher in the cheese samples from milk that was produced by cows reared in modern facilities and fed on total mixed rations, whether with or without silage [21] than by cows reared in the traditional system. Both PC varied during lactation, but in opposite directions: PC1 decreased curvilinearly, while PC2 increased linearly.

The third PC was characterized by a slightly lower intra-herd heritability than PC1 and PC2 (7.1%), and much lower across-herd heritability (3.3%) due to the large effect of herd/sampling-processing date on this component of cheese flavour (Table 5). It is worth noting that this sizeable environmental variability is not due to the dairy system but to the large variability among herds within each dairy system [21]. This PC, which explains less than 9% of the total volatile fingerprint variation, was found to increase with daily milk yield of the cow, but was not affected by parity and DIM. The fourth PC, which explained almost 8% of the total cheese volatile fingerprint, was the most heritable (${\text{h}}_{\text{AH}}^{2}$ = 8.6% and ${\text{h}}_{\text{IH}}^{2}$ = 10.2%). This PC was not much affected by dairy system or individual herd, but was found to increase during lactation [21]. Among the other PC, PC5, PC6 and PC9 were characterized by a low heritability (<6%) and PC7, PC8 and PC10 had heritabilities that ranged from 7.1 to 9.8% and were intermediate between those of PC1 and PC2 and those of PC4 (Table 5). To our knowledge, this is the first report on heritability estimates for phenotypes that describe the profile of volatile compounds in cheese. As discussed above, slightly more than one third of the spectrometry peaks had estimated heritabilities that were similar to those of the three PC of the volatile fingerprint with low heritabilities, about one third of the spectrometry peaks had estimated heritabilities similar to those of the other seven PC, and about a quarter had estimated heritabilities that were higher than those of the most heritable PC (PC4).

Given that no other data are available in the literature, it was interesting to compare the heritability of the PC of the volatile fingerprint of the model cheeses with the heritability of other traits that were studied in the same project with the same cows, or at the population level with the same breed and in the same area. Estimated heritability of daily milk yield (18.2%) [20, 38] in individual cows was about double that of most of the cheese VOC PC, while the estimated heritability reported by Cecchinato et al. [39] was similar to that of the PC with the highest heritability. Regarding milk quality, heritabilities of fat content (12.2%) and SCS (9.6%) [38] were similar to those of the PC of the cheese volatile fingerprint with the highest heritability, while milk protein (28%), casein (28%), casein number (i.e. the ratio between casein and total protein) (15.1%), lactose (17.0%) and urea (35.6%) were much more heritable at both the experimental and population levels. The estimated heritabilities of the detailed fatty acid profile of the milk samples were in the same range as those of the PC of the volatile fingerprint of cheeses obtained from the same milk, with the exception of the saturated odd-numbered fatty acids and a few others [40]. A possible explanation for such similar ranges of heritabilities could be related to the origin of the VOC, since many molecules may be produced from milk fat via different biosynthetic pathways [3]. For example, beta-oxydation and decarboxylation of milk fat produce methyl ketones and secondary alcohols, and esterification of hydroxy fatty acids produces lactones. Fatty acids can also react with alcohol groups to form esters such as ethyl butanoate and ethyl hexanoate, which are correlated with PC1.

As for the cheese-making process, the traditional milk coagulation properties were also much more heritable than the PC of the cheese volatile fingerprint, with the exception of curd firmness recorded 45 min after rennet addition [41]. Modelling of curd firming [42] in the same milk samples yielded estimated heritabilities that were higher than those of the PC of the cheese volatile fingerprint for rennet coagulation time and for the curd firming instant rate constant, and that were of the same magnitude as those for potential curd firmness and the syneresis instant rate constant [43].

The technological traits (three cheese yields and four milk nutrient recoveries in the curd) measured in the fresh model cheeses were also much more heritable than the PC of the volatile fingerprint of the same model cheeses after 2 months of ripening [20]. The same traits predicted by Fourier transform infrared spectrometry using the calibration proposed by Ferragina et al. [44] on the same milk samples [45] and at the population level [39] were characterized by heritability estimates of the same size.

In summary, the PC of the volatile compound fingerprints of cheeses obtained by using the PTR-ToF-MS procedure are not only heritable, their heritability estimates are similar to those of several milk quality traits (fat content, content in many fatty acids, and SCS) and of some coagulation properties (potential curd firmness and syneresis instant rate constant) that are already selected for or for which genetic selection has been proposed. How can animal genetic characteristics affect cheese VOC has never been studied. However, it should be emphasized that the majority of VOC in ripened cheese originate from the breakdown of fresh cheese components by (1) milk native enzymes produced by the cow, or (2) enzymatic activity of cheese micro-organisms.

For example, proteolysis releases amino acids via the Strecker reaction, which are the precursors of a wide variety of volatile compounds including aldehydes, such as 2-methylbutanal, 3-methylbutanal, hexanal and nonanal, that may be responsible for green and herbaceous aromas in cheese [13] and that are correlated with PC (see Additional file 2: Table S2). The presence and the activity of milk native enzymes in relation to cheese flavour and VOC need to be further studied, but it is known that some of these activities are under the genetic control of the lactating cow. Lipoprotein lipase (LPL), which has been well described in humans, has the potential to hydrolyse the greater part of milk fats, but this is prevented by the membrane of the fat globule [46]. Also, the casein cleavage by plasmin is a well-known reaction that leads to particular sensory characteristics of cheese depending on the milk plasmin activity [47]. Hydrolysis of lactoproteins by enzymes varies with the presence of polymorphisms that induce amino acid substitutions or deletions that change the site of cleavage of the enzymes, thereby generating further modifications of the cheese characteristics. For example, the cleavage of β-casein by plasmin differs greatly between the β-casein variants A1 and C [48, 49]. However, the growth and activity of the micro-organisms present in the milk may also be directly related to milk components with anti-microbial activity, such as lactoferrin, which has been shown to be heritable [50]. Another hypothesis is that very indirect relationships may occur between the technological properties of milk, such as curd firming and syneresis, i.e., water expulsion from the curd [51] and the growth and activity of micro-organisms in cheeses, and the main compounds of milk, such as caseins. Indeed, the content in milk caseins and their genetic variants drive coagulation and draining kinetics and may modify the water content of fresh cheese [52], which, in turn, may modify the growth or activity of micro-organisms during cheese ripening. Other similar indirect effects may also occur for other milk compounds (with variable heritabilities), such as soluble proteins, urea, SCS, carotenoids, etc.

### Phenotypic, genetic, herd/sampling-processing date and residual correlations among VOC

**Fig. 2:**
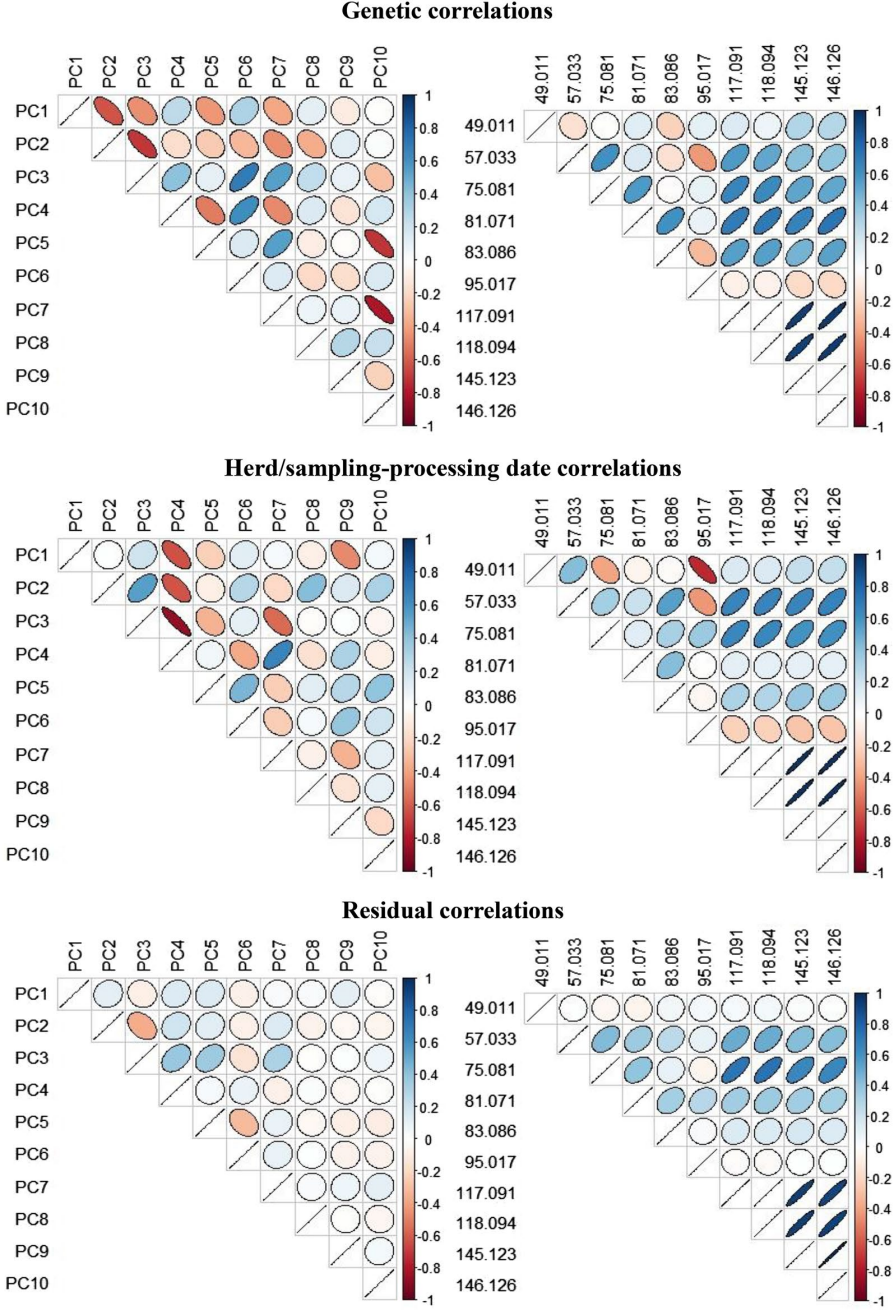
Genetic, herd/sampling-processing date and residual correlations among the first ten principal components and among the ten identified individual VOC with the highest heritability. Correlations among the first ten principal components (*left triangles*) and among the ten individual identified VOC (*right triangles*); all estimates (expressed as the mean of the marginal posterior distribution of the parameter) ranged from no correlation (*uncoloured circles*) to high correlations (*thin*, *dark-coloured ovals*); negative correlations: *reddish ovals* from *top left* to *bottom right*; positive correlations: *bluish ovals* from *top right* to *bottom left*

Figure 2 shows that, unlike the PC, the correlations between the ten VOC with the highest heritabilities varied, but were generally positive. The residual correlations were often moderate to high and positive. Only the peaks relative to methanethiol (theoretical mass *m*/*z* 49.011) and methyldisulfanylmethane (theoretical mass *m*/*z* 95.017) had very low residual correlations with the other eight VOC. These two VOC also had negative genetic and herd/sampling-processing date correlations with the others, which were generally positively correlated with each other. As discussed above, microorganisms are considered to be the key agents in the production of these volatile compounds in ripened cheese. Analysis of individual VOC concentrations is quantitative, thus it is logical that an increase in the global quantity of VOC in the cheese would result in an increase in most of the individual VOC, and especially those with the highest concentrations and therefore in positive phenotypic correlations with each other.

It is likely that methanethiol and methyldisulfanylmethane are determined by genetic and/or herd pathways that differ from those characterizing the global quantity of cheese odorants, which could explain the residual independence from other VOC. The reasons for the negative genetic and herd correlations need to be investigated in future research.

### Phenotypic, genetic, herd/sampling-processing date and residual correlations among PC

As expected with PC, the phenotypic correlations among PC were always close to 0 (−0.02 to 0.02) and the 0 value was always included in their HPD 95% (data not shown). These are the results from the multivariate data treatment that generates a few variables, either uncorrelated or with a low level of correlation, which may be desirable indicators of the volatile compound fingerprint of cheese. However, this phenotypic independence is the result of additive genetic, herd/sampling-processing date and residual correlations, which are sometimes very different from zero but opposite in sign. Figure 2 reports the genetic, herd/sampling-processing date and residual correlations among the first ten PC of the volatile compound fingerprint of cheese.

For example, the first three PC, which together explain about half of the variability of all the 240 spectrometry peaks obtained with PTR-ToF-MS, are negatively correlated with each other from a genetic point of view (Fig. 2). However, PC2 and PC3 were positively correlated for herd/sampling-processing date but had a low negative residual correlation. Other high additive genetic correlations were found between PC3 and PC6 (positive), and between PC10 and both PC5 and PC7 (negative). Some high correlations between herd-sampling and processing date were found among the ten PC, while the residual correlations were generally much lower (Fig. 2).

The correlations between PC in 60-day old cheeses could be the result of many different (and independent) metabolic pathways (i.e., lipolysis vs. proteolysis) that involve many ripening agents. Thus, correlations between PC seem to present a more qualitative picture (proportions among groups) of the volatile compound fingerprint of cheese, while the correlations between individual spectrometry peaks describe the quantitative relationships among them. Since these results cannot be compared with the literature because of lack of data, further research on these correlations is necessary to assess their importance and reveal the significance of these new phenotypes, especially in relation to the sensorial properties of cheeses. Moreover, research on the various herd and animal factors that affect the PC [21] and their correlations with milk quality traits would help to better characterize cheese flavour.

### Phenotypic, genetic, herd/sampling-processing date and residual correlations of VOC and PC with milk quality traits

**Fig. 3:**
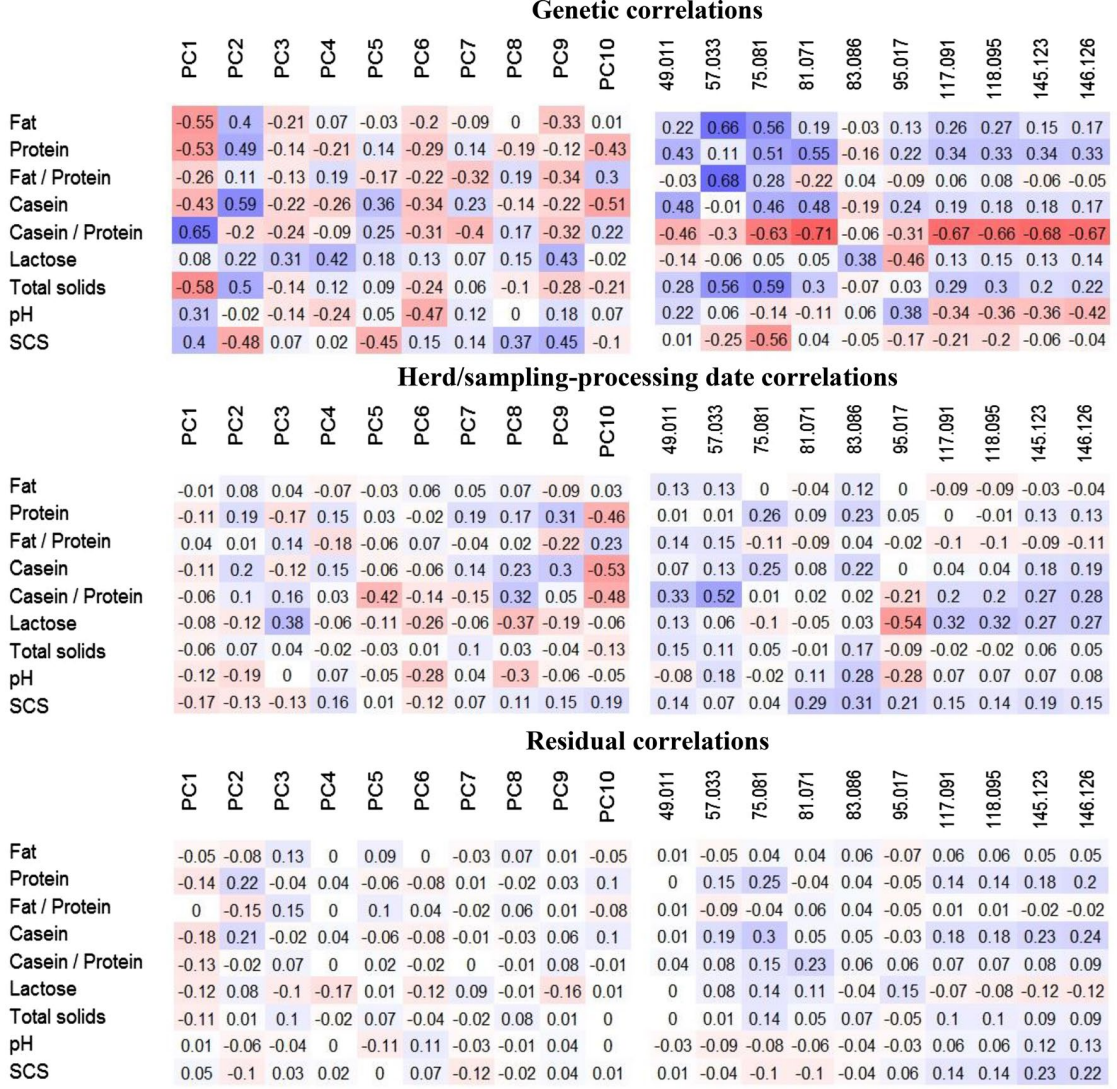
Genetic, herd/sampling-processing date and residual correlations between the first ten principal components and the ten identified individual VOC with milk quality traits. Correlations between the first ten principal components (*left squares*) and milk quality traits, and between the ten identified individual VOC (*right squares*) with the highest heritability estimates (expressed as means of the marginal posterior distribution of the parameter) and milk quality traits, ranging from *bright red* for highly negative correlations to *no colour* for uncorrelated traits, and *bright blue* for highly positive correlations

Finally, we analyzed the dependency of cheese VOC and their PC on the quality traits of the milk used for cheese making. Figure 3 shows that there are positive and negative correlation coefficients that are small at the residual level, low to moderate at the herd level and low to high at the genetic level.

We did not find any PC or VOC with patterns that clearly differed from the others. However, it is clear that some milk quality traits have a more marked effect on the ten VOC with the highest heritability. In particular, contents of fat, protein, casein and total solids in milk are generally positively correlated with all VOC, which indicates that genetic factors associated with more concentrated milk are also responsible for an increase in the content of VOC in the resulting cheese. These VOC with the highest heritability seem to reflect the initial composition (fat and casein contents, and therefore the content of VOC precursors) of the raw whole milk used in cheese making and could be considered quantitative elements.

Regarding the casein number, all the genetic correlations are negative except for two highly negative correlations. Since both the numerator and denominator of the ratio are generally positively correlated with VOC concentration, this could mean that the genetic factors responsible for an increase in the ratio between caseins and whey protein could differ from those related to the increase in total protein and casein and, thus, could be responsible for a decrease in VOC concentration.

Based on Fig. 3, milk pH and SCS may also exert some negative effects on cheese VOC, which should also be further studied.

## Conclusions

In this work, we report the first estimated genetic parameters of the cheese volatile compound profile in dairy cattle. Although the sample size was rather limited, our results are very original and show, as expected, that the large majority of the 240 spectrometry peaks obtained with PTR-ToF-MS were characterized by heritability estimates that were similar to those obtained for the PC extracted from them, while about one sixth of these 240 peaks were characterized by higher heritabilities, which were similar to those for daily milk yield and several other milk and cheese traits. Only a small proportion of the peaks had a very low heritability. Heritability estimates for the ten PC were low, although for seven of them they were similar to those for other milk traits, such as fat content, many fatty acid percentages, SCS and some curd firming model parameters. Variability due to herd-sampling-processing date varied greatly among the PC. Although the phenotypic correlations among the ten PC were, as expected, close to 0, there were some sizeable genetic correlations, in particular those among herd/sampling-processing date, while the residual correlations were generally lower. The most heritable VOC and PC of cheeses had variable and generally positive genetic, herd and residual correlations with each other. The correlations between selected VOC and PC of cheeses with quality traits of milk before cheese making were also variable and were generally higher for genetic correlations, intermediate for herd correlations and lower for residual correlations. Our results demonstrate the existence of exploitable genetic variation in the factors related to cheese volatile profiles that are potentially useful for improving the flavour of cheese. This study opens new avenues of research for the characterization of the relationships between individual peaks or PC and the sensory profile of cheese, and for the study of their use for indirect prediction using infrared technology.

## Additional files

**Additional file 1: Table S1.** Average concentrations of spectrometry peaks and coefficients of variation (CV, %) from PTR-ToF-MS analysis of 1075 cheese samples, together with their phenotypic ($\upsigma_{\text{P}}$), residual ($\upsigma_{\text{E}}$), herd ($\upsigma_{\text{H}}$) and additive genetic ($\upsigma_{\text{A}}$) SD and intra-herd heritability (h^2^). ^1^Data expressed in natural log-transformed (ln) parts per billion by volume; ^2^Mean = mean of the marginal posterior density of the parameters; ^3^PSD = posterior standard deviation.

**Additional file 2: Table S2. **Measured and theoretical mass, sum formula and tentative identification of the five PTR-ToF-MS peaks that have the highest phenotypic correlation (r) with each PC. Highest correlation coefficients between the first ten PC and the volatile compounds tentatively attributed to specific spectrometric fragments of PTR-ToF-MS spectra

## References

[1] Molimard P, Spinnler HE (1996). Review: compounds involved in the flavor of surface mold-ripened cheeses: origins and properties. J Dairy Sci.

[2] Bellesia F, Pinetti A, Pagnoni UM, Rinaldi R, Zucchi C, Caglioti L (2003). Volatile components of Grana Parmigiano-Reggiano type hard cheese. Food Chem.

[3] McSweeney PL, Sousa MJ (2000). Biochemical pathways for the production of flavour compounds in cheeses during ripening: a review. Lait.

[4] Fox PF, Wallace JM (1997). Formation of flavor compounds in cheese. Adv Appl Microbiol.

[5] Drake SL, Gerard PD, Drake MA (2008). Consumer preferences for mild Cheddar cheese flavors. J Food Sci.

[6] Liggett RE, Drake MA, Delwiche JF (2008). Impact of flavor attributes on consumer liking of Swiss cheese. J Dairy Sci.

[7] Martin B, Verdier-Metz I, Buchin S, Hurtaud C, Coulon JB (2005). How do the nature of forages and pasture diversity influence the sensory quality of dairy livestock products?. Anim Sci.

[8] Coppa M, Verdier-Metz I, Ferlay A, Pradel P, Didienne R, Farruggia A (2011). Effect of different grazing systems on upland pastures compared with hay diet on cheese sensory properties evaluated at different ripening times. Int Dairy J.

[9] Romanzin A, Corazzin M, Piasentier E, Bovolenta S (2013). Effect of rearing system (mountain pasture vs. indoor) of Simmental cows on milk composition and Montasio cheese characteristics. J Dairy Res.

[10] Bittante G, Cecchinato A, Cologna N, Penasa M, Tiezzi F, De Marchi M (2011). Factors affecting the incidence of first-quality wheels of Trentingrana cheese. J Dairy Sci.

[11] Bittante G, Cologna N, Cecchinato A, De Marchi M, Penasa M, Tiezzi F (2011). Monitoring of sensory attributes used in the quality payment system of Trentingrana cheese. J Dairy Sci.

[12] LeQuéré J, Fox PF, McSweeney PLH, Cogan TM, Guinee TP (2004). Cheese flavour: instrumental techniques. Cheese—chemistry, physics and microbiology.

[13] Cornu A, Rabiau N, Kondjoyan N, Verdier-Metz I, Pradel P, Tournayre P (2009). Odour-active compound profiles in Cantal-type cheese: effect of cow diet, milk pasteurization and cheese ripening. Int Dairy J.

[14] Carunchia Whetstine ME, Drake MA, Nelson BK, Barbano DM (2006). Flavor profiles of full-fat and reduced-fat cheese and cheese fat made from aged cheddar with the fat removed using a novel process. J Dairy Sci.

[15] Delgado FJ, González-Crespo J, Cava R, Ramírez R (2011). Formation of the aroma of a raw goat milk cheese during maturation analysed by SPME–GC–MS. Food Chem.

[16] Thomsen M, Gourrat K, Thomas-Danguin T, Guichard E (2014). Multivariate approach to reveal relationships between sensory perception of cheeses and aroma profile obtained with different extraction methods. Food Res Int.

[17] Valdivielso I, Albisu M, de Renobales M, Barron LJR (2016). Changes in the volatile composition and sensory properties of cheeses made with milk from commercial sheep flocks managed indoors, part-time grazing in valley, and extensive mountain grazing. Int Dairy J.

[18] Bergamaschi M, Aprea E, Betta E, Biasioli F, Cipolat-Gotet C, Cecchinato A (2015). Effects of dairy system, herd within dairy system, and individual cow characteristics on the volatile organic compound profile of ripened model cheeses. J Dairy Sci.

[19] Cipolat-Gotet C, Cecchinato A, De Marchi M, Bittante G (2013). Factors affecting variation of different measures of cheese yield and milk nutrient recovery from an individual model cheese-manufacturing process. J Dairy Sci.

[20] Bittante G, Cipolat-Gotet C, Cecchinato A (2013). Genetic parameters of different measures of cheese yield and milk nutrient recovery from an individual model cheese-manufacturing process. J Dairy Sci.

[21] Bergamaschi M, Biasioli F, Cappellin L, Cecchinato A, Cipolat-Gotet C, Cornu A (2015). Proton transfer reaction time-of-flight mass spectrometry: a high-throughput and innovative method to study the influence of dairy system and cow characteristics on the volatile compound fingerprint of cheeses. J Dairy Sci.

[22] Sturaro E, Marchiori E, Cocca G, Penasa M, Ramanzin M, Bittante G (2013). Dairy systems in mountainous areas: farm animal biodiversity, milk production and destination, and land use. Livest Sci.

[23] Ali AKA, Shook GE (1980). An optimum transformation for somatic cell concentration in milk. J Dairy Sci.

[24] Fabris A, Biasioli F, Granitto PM, Aprea E, Cappellin L, Schuhfried E (2010). PTR-TOF-MS and data-mining methods for rapid characterisation of agro-industrial samples: influence of milk storage conditions on the volatile compounds profile of Trentingrana cheese. J Mass Spectrom.

[25] Cappellin L, Biasioli F, Schuhfried E, Soukoulis C, Märk TD, Gasperi F (2011). Extending the dynamic range of proton transfer reaction time-of-flight mass spectrometers by a novel dead time correction. Rapid Commun Mass Spectrom.

[26] Lindinger W, Hansel A, Jordan A (1998). On-line monitoring of volatile organic compounds at pptv levels by means of proton-transfer-reaction mass spectrometry (PTR-MS) medical applications, food control and environmental research. Int J Mass Spectrom Ion Process.

[27] Gelfand AE, Smith AF (1990). Sampling-based approaches to calculating marginal densities. J Am Stat Assoc.

[28] Geweke J, Berger JO, Bernardo JM, Dawid AP, Smith AFM (1992). Evaluating the accuracy of sampling-based approaches to the calculation of posterior moments. Bayesian statistics.

[29] Gelman A, Rubin DB (1992). Inference from iterative simulation using multiple sequences. Stat Sci.

[30] Othmane MH, Carriedo JA, San Primitivo F, Fuente LDL (2002). Genetic parameters for lactation traits of milking ewes: protein content and composition, fat, somatic cells and individual laboratory cheese yield. Genet Sel Evol.

[31] Rosati A, Van Vleck LD (2002). Estimation of genetic parameters for milk, fat, protein and mozzarella cheese production for the Italian river buffalo *Bubalus bubalis* population. Livest Prod Sci.

[32] Ikonen T, Morri S, Tyrisevä A, Ruottinen O, Ojala M (2004). Genetic and phenotypic correlations between milk coagulation properties, milk production traits, somatic cell count, casein content, and pH of milk. J Dairy Sci.

[33] Carpino S, Mallia S, La Terra S, Melilli C, Licitra G, Acree T (2004). Composition and aroma compounds of Ragusano cheese: native pasture and total mixed rations. J Dairy Sci.

[34] Horne J, Carpino S, Tuminello L, Rapisarda T, Corallo L, Licitra G (2005). Differences in volatiles, and chemical, microbial and sensory characteristics between artisanal and industrial Piacentinu Ennese cheeses. Int Dairy J.

[35] Viallon C, Verdier-Metz I, Denoyer C, Pradel P, Coulon JB, Berdagué JL (1999). Desorbed terpenes and sesquiterpenes from forages and cheeses. J Dairy Res.

[36] Cornu A, Kondjoyan N, Martin B, Verdier-Metz I, Pradel P, Berdagué J (2005). Terpene profiles in Cantal and Saint-Nectaire-type cheese made from raw or pasteurised milk. J Sci Food Agric.

[37] Bugaud C, Buchin S, Coulon JB, Hauwuy A, Dupont D (2001). Influence of the nature of alpine pastures on plasmin activity, fatty acid and volatile compound composition of milk. Lait.

[38] Cecchinato A, Ribeca C, Chessa S, Cipolat-Gotet C, Maretto F, Casellas J (2014). Candidate gene association analysis for milk yield, composition, urea nitrogen and somatic cell scores in Brown Swiss cows. Animal.

[39] Cecchinato A, Chessa S, Ribeca C, Cipolat-Gotet C, Bobbo T, Casellas J (2015). Genetic variation and effects of candidate-gene polymorphisms on coagulation properties, curd firmness modeling and acidity in milk from Brown Swiss cows. Animal.

[40] Pegolo S, Cecchinato A, Casellas J, Conte G, Mele M, Schiavon S (2015). Genetic and environmental relationships of detailed milk fatty acids profile determined by gas chromatography in Brown Swiss cows. J Dairy Sci.

[41] Cecchinato A, Cipolat-Gotet C, Casellas J, Penasa M, Rossoni A, Bittante G (2013). Genetic analysis of rennet coagulation time, curd-firming rate, and curd firmness assessed over an extended testing period using mechanical and near-infrared instruments. J Dairy Sci.

[42] Bittante G, Contiero B, Cecchinato A (2013). Prolonged observation and modelling of milk coagulation, curd firming, and syneresis. Int Dairy J.

[43] Cecchinato A, Albera A, Cipolat-Gotet C, Ferragina A, Bittante G (2015). Genetic parameters of cheese yield and curd nutrient recovery or whey loss traits predicted using Fourier-transform infrared spectroscopy of samples collected during milk recording on Holstein, Brown Swiss, and Simmental dairy cows. J Dairy Sci.

[44] Ferragina A, Cipolat-Gotet C, Cecchinato A, Bittante G (2013). The use of Fourier-transform infrared spectroscopy to predict cheese yield and nutrient recovery or whey loss traits from unprocessed bovine milk samples. J Dairy Sci.

[45] Bittante G, Ferragina A, Cipolat-Gotet C, Cecchinato A (2014). Comparison between genetic parameters of cheese yield and nutrient recovery or whey loss traits measured from individual model cheese-making methods or predicted from unprocessed bovine milk samples using Fourier-transform infrared spectroscopy. J Dairy Sci.

[46] Deeth HC (2006). Lipoprotein lipase and lipolysis in milk. Int Dairy J.

[47] Coulon JB, Delacroix-Buchet A, Martin B, Pirisi A (2004). Relationships between ruminant management and sensory characteristics of cheeses: a review. Lait.

[48] Marie C, Delacroix-Buchet A (1994). Comparaison des variants A et C de la caséine β des laits de vaches tarentaises en modèle fromager de type Beaufort. II. Protéolyse et qualité des fromages. Lait.

[49] Papoff C, Delacroix-Buchet A, Le Bars D, Campus R, Vodret A (1995). Hydrolysis of bovine β-casein C by plasmin. Ital J Food Sci.

[50] Soyeurt H, Colinet F, Arnould V, Dardenne P, Bertozzi C, Renaville R (2007). Genetic variability of lactoferrin content estimated by mid-infrared spectrometry in bovine milk. J Dairy Sci.

[51] Law B, Law BA, Tamime AY (2010). Cheese-ripening and cheese flavour technology. Technology of Cheesemaking.

[52] Caroli AM, Chessa S, Erhardt GJ (2009). Invited review: milk protein polymorphisms in cattle: effect on animal breeding and human nutrition. J Dairy Sci.

