# Supplementary material for: From cow to cheese: genetic parameters of the flavour fingerprint of cheese investigated by direct-injection mass spectrometry (PTR-ToF-MS)
Source: Genet Sel Evol. 2016 Nov 16;48:89. doi: 10.1186/s12711-016-0263-4 (PMC5112698; doi:10.1186/s12711-016-0263-4)
Supplement: Supplementary file 1 — Additional file 1: Table S1. Average concentrations of spectrometry peaks and coefficients of variation (CV, %) from PTR-ToF-MS analysis of 1075 cheese samples, together with their phenotypic ($\upsigma_{\text{P}}$σP), residual ($\upsigma_{\text{E}}$σE), herd ($\upsigma_{\text{H}}$σH) and additive genetic ($\upsigma_{\text{A}}$σA) SD and intra-herd heritability (h2). 1Data expressed in natural log-transformed (ln) parts per billion by volume; 2Mean = mean of the marginal posterior density of the parameters; 3PSD = posterior standard deviation. [file 12711_2016_263_MOESM1_ESM.pdf]

m/z	ln ppb$_v$[1]	CV, %	σ_p	σ_E	σ_H	σ_G	Heritability	
							Mean[2]	PSD[3]
33.034	9.24	6.6	1.022	0.429	0.921	0.113	0.065	0.009
34.037	5.11	10.8	1.023	0.407	0.930	0.124	0.085	0.010
34.995	4.47	15.2	0.990	0.887	0.388	0.206	0.051	0.032
39.023	7.64	9.2	1.005	0.826	0.498	0.282	0.105	0.053
40.027	4.87	11.9	1.008	0.855	0.470	0.251	0.079	0.045
41.039	10.35	4.5	1.000	0.776	0.593	0.214	0.071	0.032
42.010	5.95	7.5	0.992	0.894	0.351	0.251	0.073	0.042
42.034	8.08	11.2	1.026	0.308	0.976	0.072	0.052	0.004
42.043	7.39	7.5	1.007	0.851	0.488	0.229	0.067	0.036
43.018	11.25	3.7	1.013	0.855	0.509	0.188	0.046	0.026
43.054	11.09	3.4	0.997	0.864	0.447	0.219	0.060	0.034
43.094	5.07	7.2	1.001	0.869	0.434	0.241	0.071	0.041
44.022	7.56	5.1	1.013	0.855	0.511	0.185	0.045	0.025
44.058	7.78	5.2	0.996	0.831	0.502	0.221	0.066	0.034
44.980	4.64	14.2	1.006	0.928	0.203	0.330	0.113	0.060
45.033	10.93	5.1	1.008	0.765	0.625	0.204	0.066	0.031
46.031	5.74	11.9	1.013	0.739	0.639	0.267	0.115	0.045
46.038	7.14	8.8	1.009	0.774	0.622	0.181	0.052	0.025
48.012	5.69	7.7	0.991	0.763	0.550	0.311	0.143	0.049
48.053	10.03	8.2	0.995	0.741	0.598	0.287	0.131	0.044
49.011	6.82	9.8	0.994	0.800	0.507	0.302	0.125	0.051
49.028	5.28	13.8	1.006	0.896	0.374	0.262	0.078	0.051
49.054	7.72	10.3	0.995	0.737	0.600	0.296	0.139	0.045
50.000	5.11	16.6	0.995	0.750	0.555	0.346	0.176	0.053
50.057	4.58	9.6	0.992	0.754	0.562	0.313	0.147	0.050
51.007	4.59	10.5	0.993	0.801	0.510	0.290	0.116	0.050
51.044	7.68	7.9	1.020	0.577	0.832	0.130	0.048	0.013
53.039	4.50	12.1	1.014	0.702	0.717	0.145	0.041	0.017
54.034	4.24	13.9	1.029	0.255	0.992	0.098	0.129	0.005
55.055	7.08	22.1	1.000	0.915	0.327	0.239	0.064	0.040
56.045	5.33	9.8	0.988	0.957	0.122	0.212	0.047	0.037
56.060	5.86	8.7	1.017	0.704	0.700	0.223	0.091	0.032
57.033	6.70	10.0	1.007	0.805	0.516	0.317	0.134	0.058
57.070	10.55	6.2	0.991	0.753	0.606	0.217	0.077	0.033
58.041	5.72	15.5	0.991	0.839	0.471	0.239	0.075	0.039
58.073	7.47	8.7	0.992	0.747	0.613	0.228	0.085	0.035
59.049	10.52	7.5	1.010	0.696	0.715	0.161	0.051	0.020
59.329	4.34	11.7	1.008	0.699	0.708	0.165	0.053	0.022
60.021	5.81	13.9	1.002	0.867	0.432	0.257	0.081	0.048
60.045	5.39	13.5	1.023	0.600	0.818	0.134	0.047	0.014
60.053	7.15	10.7	1.010	0.696	0.714	0.162	0.051	0.021
61.028	11.04	4.4	1.009	0.867	0.478	0.194	0.048	0.027
61.062	7.19	5.8	0.996	0.840	0.482	0.231	0.070	0.036

62.032	7.38	6.2	1.010	0.867	0.482	0.193	0.047	0.027
62.068	4.40	6.9	0.993	0.828	0.464	0.291	0.110	0.049
63.027	7.10	13.3	1.028	0.594	0.824	0.161	0.068	0.018
63.044	8.02	6.1	1.009	0.812	0.570	0.183	0.048	0.026
64.031	4.48	13.7	1.026	0.618	0.799	0.180	0.078	0.022
64.048	4.82	8.8	1.006	0.794	0.572	0.235	0.080	0.040
65.018	5.37	13.4	1.007	0.866	0.476	0.194	0.048	0.026
66.063	8.61	10.0	0.995	0.766	0.567	0.286	0.122	0.044
67.058	5.65	7.8	0.998	0.896	0.396	0.191	0.043	0.030
67.065	6.75	13.3	0.998	0.767	0.570	0.288	0.124	0.044
68.067	4.29	7.7	0.992	0.794	0.510	0.305	0.129	0.050
69.033	4.35	8.8	1.008	0.799	0.583	0.191	0.054	0.027
69.058	4.58	18.5	1.007	0.924	0.337	0.217	0.052	0.035
69.070	8.43	6.9	1.006	0.871	0.459	0.209	0.054	0.031
70.064	5.13	9.8	1.017	0.674	0.742	0.171	0.061	0.021
70.078	6.89	9.4	1.010	0.756	0.643	0.190	0.059	0.025
71.049	9.50	6.3	1.009	0.888	0.425	0.224	0.060	0.038
71.086	10.38	4.5	0.991	0.835	0.496	0.199	0.054	0.029
72.053	6.53	8.6	1.009	0.892	0.420	0.217	0.056	0.038
72.089	7.54	5.9	0.993	0.829	0.508	0.202	0.056	0.030
73.027	5.50	12.2	1.006	0.908	0.351	0.254	0.072	0.050
73.051	6.57	18.6	1.011	0.981	0.127	0.212	0.044	0.036
73.065	9.88	6.3	1.011	0.838	0.543	0.154	0.032	0.019
74.034	4.34	12.0	1.004	0.863	0.459	0.231	0.067	0.037
74.051	4.49	13.8	1.006	0.970	0.101	0.245	0.060	0.044
74.069	6.85	8.7	1.010	0.839	0.542	0.155	0.033	0.020
75.027	5.47	17.6	1.010	0.954	0.096	0.320	0.101	0.060
75.044	8.22	8.2	1.025	0.490	0.888	0.146	0.081	0.013
75.080	7.70	14.8	0.990	0.763	0.554	0.302	0.136	0.048
76.047	5.26	10.3	1.023	0.469	0.903	0.110	0.052	0.009
76.084	5.12	15.5	0.987	0.769	0.534	0.313	0.142	0.052
77.060	6.43	10.5	1.013	0.760	0.649	0.163	0.044	0.021
78.001	4.23	21.0	0.999	0.944	0.232	0.230	0.056	0.037
79.040	8.53	7.2	1.010	0.900	0.424	0.178	0.038	0.024
79.055	6.87	23.3	1.006	0.964	0.186	0.220	0.050	0.038
79.075	6.17	10.4	0.991	0.835	0.477	0.241	0.077	0.039
80.046	5.47	8.4	1.009	0.895	0.432	0.170	0.035	0.024
80.058	4.66	20.0	1.004	0.958	0.218	0.208	0.045	0.034
80.991	6.17	10.3	1.004	0.783	0.596	0.198	0.060	0.028
81.039	4.63	8.2	1.010	0.843	0.534	0.153	0.032	0.019
81.061	4.87	10.7	1.018	0.683	0.730	0.193	0.074	0.028
81.070	5.37	8.6	1.021	0.669	0.692	0.340	0.206	0.056
82.945	4.48	15.6	1.028	0.220	1.001	0.087	0.135	0.004
82.988	5.20	11.2	1.001	0.789	0.588	0.183	0.051	0.025
83.052	4.97	10.7	1.009	0.798	0.568	0.241	0.084	0.036
83.071	7.44	12.8	0.996	0.787	0.536	0.291	0.120	0.046

83.086	6.13	11.3	0.998	0.825	0.453	0.333	0.140	0.056
84.075	4.41	13.1	0.988	0.839	0.420	0.309	0.119	0.052
84.079	4.25	13.4	1.004	0.938	0.248	0.258	0.070	0.046
84.942	4.29	14.7	1.027	0.220	1.001	0.076	0.106	0.003
85.029	4.46	8.9	1.005	0.910	0.320	0.283	0.088	0.051
85.065	6.60	9.1	1.001	0.924	0.342	0.177	0.035	0.025
85.101	6.66	8.3	1.007	0.725	0.654	0.245	0.102	0.038
86.072	5.00	14.1	0.992	0.829	0.483	0.249	0.083	0.041
86.105	4.54	9.5	1.007	0.728	0.649	0.250	0.106	0.041
87.044	7.20	11.0	1.010	0.890	0.425	0.217	0.056	0.036
87.080	10.42	10.4	1.000	0.791	0.577	0.203	0.061	0.029
88.052	5.22	9.6	1.015	0.840	0.536	0.192	0.050	0.029
88.084	7.61	12.4	1.001	0.789	0.569	0.238	0.083	0.035
89.060	10.76	4.1	1.010	0.818	0.553	0.210	0.062	0.031
90.063	7.69	5.6	1.010	0.818	0.554	0.207	0.060	0.030
91.051	6.52	22.4	1.007	0.906	0.375	0.231	0.061	0.039
91.059	8.47	9.5	1.009	0.936	0.211	0.313	0.101	0.057
92.061	6.64	14.6	1.023	0.492	0.885	0.150	0.085	0.016
93.037	7.78	10.0	1.007	0.947	0.261	0.220	0.051	0.037
93.069	8.96	18.0	1.009	0.716	0.693	0.160	0.048	0.022
93.090	10.11	13.5	0.996	0.764	0.566	0.294	0.129	0.048
93.181	4.19	12.5	0.998	0.871	0.399	0.281	0.094	0.050
93.432	4.54	10.0	0.998	0.759	0.542	0.354	0.179	0.062
94.039	5.43	16.5	1.009	0.971	0.161	0.221	0.049	0.038
94.074	6.64	20.4	1.021	0.437	0.913	0.135	0.087	0.013
94.095	6.92	19.8	0.996	0.767	0.561	0.298	0.131	0.046
95.004	4.72	13.8	1.021	0.567	0.825	0.199	0.110	0.024
95.017	5.04	14.1	1.013	0.847	0.415	0.370	0.161	0.064
95.034	5.22	12.3	1.005	0.942	0.202	0.286	0.085	0.055
95.049	4.98	11.3	1.024	0.367	0.946	0.135	0.119	0.012
95.081	4.93	13.5	1.020	0.430	0.920	0.093	0.045	0.007
95.096	5.44	18.1	0.991	0.766	0.534	0.331	0.157	0.055
96.961	6.15	10.0	1.004	0.793	0.588	0.179	0.048	0.024
97.060	4.87	7.1	0.998	0.773	0.610	0.166	0.044	0.021
97.101	6.23	9.6	1.006	0.901	0.364	0.260	0.077	0.045
98.105	4.43	10.5	1.005	0.919	0.333	0.232	0.060	0.039
98.959	5.77	10.5	1.002	0.783	0.597	0.186	0.054	0.026
99.039	5.00	7.4	0.997	0.922	0.294	0.242	0.065	0.043
99.081	6.93	7.6	0.992	0.908	0.324	0.232	0.061	0.040
99.121	5.19	13.1	1.011	0.815	0.570	0.181	0.047	0.025
100.084	4.78	8.5	0.994	0.903	0.324	0.260	0.077	0.044
100.954	4.54	11.3	1.003	0.788	0.596	0.172	0.046	0.022
101.060	6.36	5.3	1.002	0.884	0.413	0.229	0.063	0.037
101.097	6.38	11.6	1.002	0.825	0.524	0.222	0.068	0.034
102.062	4.35	8.1	1.007	0.897	0.395	0.230	0.062	0.038
102.099	4.52	11.1	1.003	0.830	0.507	0.246	0.081	0.038

103.075	7.76	8.0	1.004	0.902	0.406	0.167	0.033	0.023
104.079	5.23	10.4	1.003	0.896	0.418	0.167	0.033	0.023
105.039	4.53	9.7	1.008	0.942	0.218	0.283	0.083	0.056
105.071	5.96	7.7	1.014	0.817	0.574	0.176	0.045	0.025
105.091	6.37	11.4	1.010	0.799	0.582	0.209	0.064	0.030
106.077	5.27	12.4	1.006	0.945	0.254	0.232	0.057	0.040
106.097	4.27	10.7	1.012	0.806	0.575	0.209	0.063	0.030
107.066	7.14	18.5	0.999	0.903	0.379	0.199	0.046	0.030
107.085	9.06	9.8	1.002	0.959	0.174	0.232	0.055	0.041
108.069	4.79	13.2	0.996	0.889	0.404	0.196	0.046	0.029
108.089	6.63	12.7	1.003	0.960	0.172	0.230	0.054	0.040
109.070	6.30	11.1	1.008	0.941	0.181	0.314	0.101	0.062
109.099	5.08	8.0	1.000	0.900	0.382	0.210	0.051	0.034
110.071	4.49	11.8	1.008	0.945	0.177	0.304	0.094	0.057
111.047	6.01	13.9	1.009	0.956	0.109	0.304	0.092	0.058
111.080	4.55	8.7	1.002	0.960	0.209	0.199	0.041	0.032
111.104	5.08	18.5	0.991	0.774	0.528	0.322	0.147	0.053
111.119	4.43	15.4	1.015	0.616	0.785	0.186	0.083	0.024
112.049	4.31	13.1	1.009	0.946	0.147	0.317	0.101	0.063
113.029	4.64	10.4	1.007	0.947	0.220	0.260	0.070	0.046
113.057	4.92	7.5	1.003	0.944	0.232	0.245	0.063	0.044
113.098	4.83	9.9	1.011	0.886	0.430	0.230	0.063	0.037
115.077	5.84	8.8	1.007	0.864	0.424	0.299	0.107	0.055
115.112	8.78	12.0	1.000	0.883	0.396	0.254	0.076	0.044
116.078	4.47	8.6	1.006	0.845	0.490	0.240	0.075	0.039
116.116	6.32	14.8	1.001	0.884	0.399	0.249	0.074	0.044
117.047	4.87	13.0	0.990	0.909	0.307	0.243	0.067	0.043
117.091	9.14	6.7	0.986	0.780	0.526	0.295	0.125	0.050
118.095	6.53	8.7	0.986	0.775	0.531	0.298	0.129	0.051
119.072	5.54	11.9	0.997	0.792	0.530	0.293	0.121	0.046
119.089	6.44	15.5	1.000	0.815	0.477	0.330	0.141	0.054
119.107	6.36	8.4	1.013	0.639	0.768	0.166	0.063	0.020
120.092	4.95	14.5	0.998	0.797	0.478	0.363	0.172	0.062
121.068	6.43	10.5	0.997	0.883	0.372	0.275	0.089	0.049
121.096	5.61	7.5	1.000	0.853	0.477	0.212	0.058	0.034
121.122	5.17	15.8	0.986	0.721	0.572	0.352	0.192	0.058
122.072	4.76	10.1	0.998	0.888	0.369	0.265	0.082	0.046
122.118	4.18	7.2	0.987	0.791	0.529	0.260	0.097	0.047
123.047	4.56	7.8	0.996	0.864	0.396	0.299	0.107	0.055
123.076	4.56	8.4	1.001	0.880	0.384	0.280	0.092	0.049
123.117	4.82	13.2	1.007	0.944	0.229	0.264	0.072	0.047
125.095	4.54	7.0	1.004	0.894	0.382	0.249	0.072	0.042
125.132	4.39	10.2	1.012	0.900	0.388	0.252	0.073	0.044
127.073	4.49	8.6	1.000	0.934	0.262	0.243	0.063	0.042
127.112	5.31	7.5	1.002	0.900	0.331	0.289	0.094	0.053
129.064	4.37	8.4	1.000	0.937	0.231	0.263	0.073	0.048

129.091	4.93	8.0	1.017	0.802	0.604	0.157	0.037	0.020
129.127	4.95	13.9	1.006	0.913	0.340	0.250	0.070	0.044
131.084	4.61	9.4	1.011	0.881	0.467	0.166	0.034	0.023
131.107	5.75	11.5	0.992	0.697	0.689	0.151	0.045	0.019
132.109	4.29	9.2	0.998	0.707	0.687	0.150	0.043	0.019
133.073	4.45	8.4	1.003	0.909	0.369	0.209	0.050	0.034
133.102	4.45	8.6	1.013	0.693	0.722	0.156	0.048	0.020
133.123	5.60	12.3	1.008	0.826	0.515	0.264	0.093	0.041
135.102	6.69	9.2	0.991	0.848	0.431	0.279	0.097	0.046
135.134	5.80	12.9	0.989	0.755	0.560	0.307	0.142	0.047
136.022	6.66	7.5	0.991	0.896	0.334	0.258	0.076	0.046
136.105	4.67	8.1	0.991	0.842	0.437	0.288	0.105	0.048
136.140	4.26	8.9	0.990	0.759	0.549	0.319	0.150	0.052
137.024	4.70	9.3	0.991	0.907	0.321	0.240	0.066	0.042
137.101	4.71	10.6	0.994	0.837	0.446	0.298	0.112	0.052
137.132	5.28	7.3	1.019	0.663	0.691	0.348	0.216	0.060
138.018	4.36	9.9	0.989	0.902	0.296	0.277	0.086	0.053
139.076	4.58	7.7	0.998	0.895	0.341	0.282	0.090	0.050
139.134	4.12	13.1	0.988	0.821	0.446	0.323	0.134	0.059
141.129	5.52	15.5	1.008	0.939	0.236	0.281	0.082	0.052
142.131	4.26	13.4	1.007	0.942	0.223	0.276	0.079	0.052
143.115	4.69	11.7	0.995	0.891	0.351	0.271	0.085	0.050
143.143	7.11	16.7	1.009	0.924	0.292	0.280	0.084	0.052
144.146	5.20	18.6	1.008	0.928	0.270	0.285	0.086	0.054
145.123	7.43	10.5	0.971	0.777	0.495	0.304	0.133	0.054
146.126	5.30	11.6	0.972	0.774	0.498	0.310	0.138	0.055
147.113	4.37	6.9	1.002	0.893	0.391	0.234	0.064	0.042
147.134	4.37	7.4	0.989	0.831	0.473	0.255	0.086	0.042
149.045	6.08	15.9	1.007	0.964	0.099	0.274	0.075	0.055
149.123	5.49	6.6	0.988	0.906	0.344	0.189	0.042	0.030
150.046	4.67	16.0	1.006	0.965	0.099	0.268	0.072	0.052
151.034	4.64	15.9	1.006	0.950	0.087	0.319	0.101	0.063
155.144	4.26	9.2	1.008	0.935	0.285	0.244	0.064	0.044
157.159	4.33	10.5	1.008	0.897	0.335	0.314	0.109	0.062
159.065	5.00	16.8	1.002	0.943	0.266	0.208	0.046	0.032
159.138	4.49	8.5	1.004	0.877	0.399	0.282	0.094	0.053
161.104	4.39	8.2	1.007	0.891	0.434	0.181	0.039	0.027
161.154	4.82	13.6	1.009	0.917	0.331	0.260	0.074	0.046
163.096	6.55	19.1	1.007	0.969	0.129	0.241	0.058	0.042
163.131	5.72	8.3	0.986	0.862	0.411	0.247	0.076	0.043
164.100	5.30	15.9	1.003	0.950	0.184	0.262	0.070	0.049
165.083	5.74	14.8	1.007	0.960	0.128	0.277	0.077	0.052
166.083	4.45	13.7	1.007	0.965	0.134	0.253	0.064	0.047
167.056	7.45	14.2	1.007	0.972	0.100	0.246	0.060	0.045
168.057	5.71	16.6	1.007	0.973	0.103	0.238	0.057	0.043
169.044	5.92	15.6	1.006	0.967	0.122	0.250	0.062	0.046

170.041	4.44	15.0	1.004	0.966	0.135	0.239	0.058	0.045
171.032	4.43	13.3	1.005	0.967	0.126	0.245	0.060	0.045
171.173	4.87	15.7	1.004	0.905	0.202	0.384	0.152	0.077
173.153	5.20	9.3	0.997	0.825	0.440	0.345	0.149	0.060
177.076	4.67	12.8	1.001	0.868	0.427	0.258	0.081	0.046
177.150	4.71	5.9	0.993	0.888	0.374	0.240	0.068	0.042
189.184	4.23	11.7	1.010	0.901	0.350	0.291	0.094	0.053
191.163	4.35	7.3	0.984	0.831	0.451	0.270	0.095	0.046
195.087	5.59	17.1	1.000	0.805	0.496	0.326	0.141	0.056
196.088	4.48	14.3	0.999	0.819	0.462	0.337	0.145	0.058
197.074	4.64	13.8	1.000	0.821	0.469	0.325	0.135	0.056
201.184	4.34	9.4	1.013	0.901	0.341	0.312	0.107	0.053
205.186	4.29	7.1	0.992	0.880	0.365	0.278	0.091	0.053
