# Supplementary material for: From cow to cheese: genetic parameters of the flavour fingerprint of cheese investigated by direct-injection mass spectrometry (PTR-ToF-MS)
Source: Genet Sel Evol. 2016 Nov 16;48:89. doi: 10.1186/s12711-016-0263-4 (PMC5112698; doi:10.1186/s12711-016-0263-4)
Supplement: Supplementary file 2 — Additional file 2: Table S2. Measured and theoretical mass, sum formula and tentative identification of the five PTR-ToF-MS peaks that have the highest phenotypic correlation (r) with each PC. Highest correlation coefficients between the first ten PC and the volatile compounds tentatively attributed to specific spectrometric fragments of PTR-ToF-MS spectra [file 12711_2016_263_MOESM2_ESM.pdf]

PC	Measured mass (*m/z*)	Sum formula	Theoretical mass (*m/z*)	r	Tentative identification
1	117.091	$C_6H_{13}O_2^+$	117.0910	-0.80	Ethyl butanoate, ethyl-2-methylpropanoate (ethyl isobutyrate), hexanoic acid
	127.112	$C_8H_{15}O^+$	127.1120	-0.73	1-Octen-3-one
	145.123	$C_8H_{17}O_2^+$	145.1229	-0.73	Ethyl hexanoate, octanoic acid
	99.081	$C_6H_{11}O^+$	99.0804	-0.69	Hexanoic acid
	71.086	$C_5H_{11}^+$	71.0855	-0.61	3-Methyl-1-butanol, 3-methyl-3-buten-1-ol, pentan-1-ol
2	69.070	$C_5H_9^+$	69.0698	-0.62	2-Methyl-1,3-butadiene (isoprene)
	115.112	$C_7H_{15}O^+$	115.1117	-0.59	Heptan-2-one
	59.049	$C_3H_7O^+$	59.0491	-0.59	Propan-2-one (acetone)
	87.080	$C_5H_{11}O^+$	87.0804	-0.56	2-Methylbutanal, 3-methylbutanal, pentan-2-one
	101.097	$C_6H_{13}O^+$	101.0961	-0.55	Hexan-1-one, hexan-2-one, hexanal
3	42.034	$C_2H_4N^+$	42.0330	-0.70	Acetonitrile
	33.034	CH_5O^+	33.0335	-0.69	Methanol
	129.127	$C_8H_{17}O^+$	129.1270	0.66	Octan-1-one
	75.044	$C_3H_7O_2^+$	75.0440	-0.61	Propanoic acid
	143.143	$C_9H_{19}O^+$	143.1430	0.60	Nonan-2-one
4	109.070	$C_2{}^{13}CH_{10}O_3N^+$	109.0760	-0.65	2,6-Dimethylpyrazine
	95.017	$C_2H_7O_2S^+$	95.0160	-0.52	Methyldisulfanylmethane (dimethyl disulphide)
	75.044	$C_3H_7O_2^+$	75.0440	0.40	Propanoic acid
	61.028	$C_2H_5O_2^+$	61.0284	0.31	Acetic acid and fragment of acetate ester
	33.034	CH_5O^+	33.0335	0.31	Methanol
5	60.021	$C_2H_4O_2^+$	60.0205	-0.61	Acetic acid
	103.075	$C_5H_{11}O_2^+$	103.0754	-0.58	3-Methylbutanoic acid (isovaleric acid), ethyl (S)-2-hydroxypropanoate (ethyl lactate), pentanoic acid (valeric acid)
	105.091	$C_5H_{13}O_2^+$	105.0910	0.55	1,2-Pentanediol
	87.080	$C_5H_{11}O^+$	87.0804	0.53	2-Methylbutanal, 3-methylbutanal, pentan-2-one
	131.107	$C_7H_{15}O_2^+$	131.1067	-0.49	Ethyl-2-methylbutanoate, ethyl-3-methylbutanoate (ethyl isovalerate), heptanoic acid
6	71.049	$C_4H_7O^+$	71.0491	0.57	Butanoic acid
	41.039	$C_3H_5^+$	41.0386	-0.56	Alkyl fragment
	57.070	$C_4H_9^+$	57.0699	-0.46	Alkyl fragment
	90.063	$C_3{}^{[13]}CH_9O_2^+$	90.0631	0.41	2-Methylpropanoic acid (isobutyric acid), butanoic acid, ethyl acetate
	89.060	$C_4H_9O_2^+$	89.0597	0.41	3-Hydroxy-2-butanone (acetoin)
7	144.146	$C_8{}^{[13]}CH_{19}O^+$	144.1460	0.43	Nonan-2-one
	143.143	$C_9H_{19}O^+$	143.1430	0.42	Nonan-2-one
	45.033	$C_2H_5O^+$	45.0335	-0.38	Ethanal (acetaldehyde)
	41.039	$C_3H_5^+$	41.0386	-0.38	Alkyl fragment

	59.049	$C_3H_7O^+$	59.0491	-0.32	Propan-2-one (acetone)
8	105.071	C_8H_{9+}	105.0700	-0.32	2-Phenylethanol/styrene
	143.143	$C_9H_{19}O^+$	143.1430	0.24	Nonan-2-one
	55.055	$C_4H_7^+$	55.0542	0.20	Butanal, heptanal, alkyl fragment
	61.028	$C_2H_5O_2^+$	61.0284	0.19	Acetic acid and fragment of acetate ester
	132.109	$C_6{}^{[13]}CH_{15}O_2^+$	132.1100	-0.18	Ethyl-2-methylbutanoate, ethyl-3-methylbutanoate (ethyl isovalerate), heptanoic acid
9	55.055	$C_4H_7^+$	55.0542	0.32	Butanal, heptanal, alkyl fragment
	71.086	$C_5H_{11}^+$	71.0855	-0.29	3-Methyl-1-butanol, 3-methyl-3-buten-1-ol, pentan-1-ol
	49.011	CH_5S^+	49.0106	-0.25	Methanethiol
	73.065	$C_4H_9O^+$	73.0648	0.25	Butan-2-one, butanal
	74.069	$C_3{}^{[13]}CH_9O^+$	73.0648	0.22	Butane-2,3-diol
10	45.033	$C_2H_5O^+$	45.0335	0.39	Ethanal (acetaldehyde)
	49.011	CH_5S^+	49.0106	-0.23	Methanethiol
	132.109	$C_6{}^{[13]}CH_{15}O_2^+$	132.1100	-0.21	Ethyl-2-methylbutanoate, ethyl-3-methylbutanoate (ethyl isovalerate), heptanoic acid
	69.070	$C_5H_9^+$	69.0698	-0.18	2-Methyl-1,3-butadiene (isoprene)
	99.081	$C_6H_{11}O^+$	99.0804	0.16	Hexanoic acid
